# Supplementary material for: The interactome of intact mitochondria by cross-linking mass spectrometry provides evidence for coexisting respiratory supercomplexes
Source: Mol Cell Proteomics. 2017 Dec 8;17(2):216–32. doi: 10.1074/mcp.RA117.000470 (PMC5795388; doi:10.1074/mcp.RA117.000470)
Supplement: Supplemental Data [file supp_RA117.000470_133922_0_supp_23978_fzffwf.zip › spectra_annotation/mito_DR_spectra_annotation/277-1-11-1-4-1.pdf]

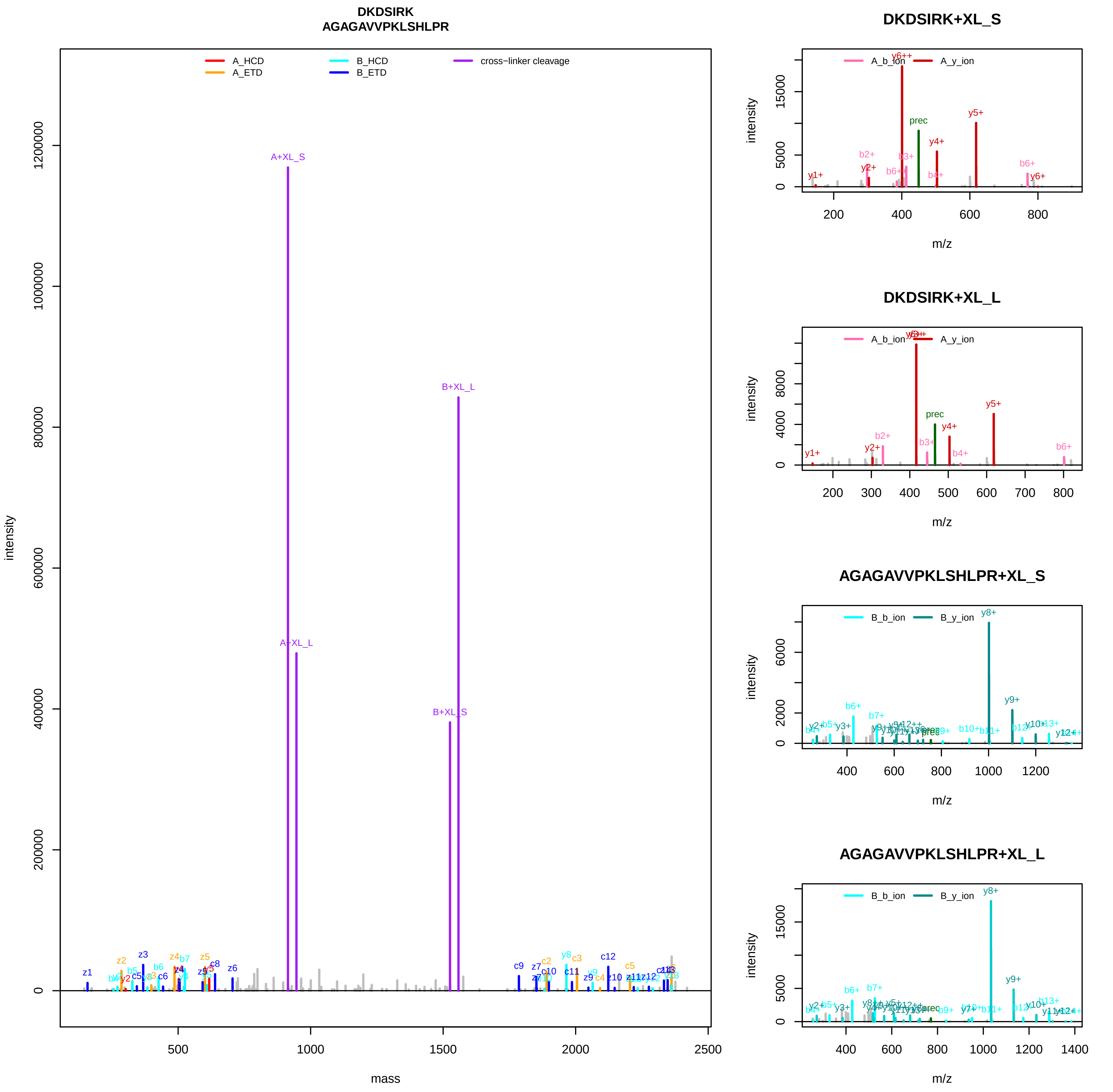
DKDSIRK
AGAGAGAVVPKLSHLPR
A_HCD
A_ETD
B_HCD
B_ETD
cross−linker cleavage
A+XL_S
A+XL_L
B+XL_L
B+XL_S
intensity
mass
0
200000
400000
600000
800000
1000000
1200000
500
1000
1500
2000
2500
DKDSIRK+XL_S
A_b_ion
A_y_ion
y6++
prec
y5+
y4+
b2+
y2+
b3+
b6+
b4+
y1+
y6+
intensity
m/z
DKDSIRK+XL_L
A_b_ion
A_y_ion
y5+
prec
y4+
b2+
y2+
b3+
b4+
y1+
b6+
intensity
m/z
AGAGAGAVVPKLSHLPR+XL_S
B_b_ion
B_y_ion
y8+
y9+
b6+
b7+
y3+
intensity
m/z
AGAGAGAVVPKLSHLPR+XL_L
B_b_ion
B_y_ion
y8+
y9+
b6+
b7+
y3+
intensity
m/z
